# Expression Signatures of Vascular Complication‐Associated Proteins in Type 2 Diabetes: A Multiomics Analysis From the FIELD Study

**DOI:** 10.1155/jdr/2756059

**Published:** 2026-07-19

**Authors:** Habib Francis, Andrzej S. Januszewski, Abubakar S. Mangani, Matthew B. O′Rourke, Michael L. H. Huang, Fahmida K. Ema, Anandwardhan A. Hardikar, Mugdha V. Joglekar, David R. Sullivan, Ronald C. W. Ma, Sanjeev Galande, Val Gebski, Michael d′Emden, R. John Simes, Alicia J. Jenkins, Mark P. Molloy, Anthony C. Keech

**Affiliations:** ^1^ NHMRC Clinical Trials Centre, Faculty of Medicine and Health, The University of Sydney, Sydney, Australia, sydney.edu.au; ^2^ Sydney Pharmacy School, The University of Sydney, Sydney, Australia, sydney.edu.au; ^3^ Bowel Cancer and Biomarker Research Laboratory, School of Medical Sciences, Kolling Institute, The University of Sydney, St Leonards, Australia, sydney.edu.au; ^4^ Baker Heart and Diabetes Institute, Melbourne, Australia, baker.edu.au; ^5^ Western Sydney University School of Medicine, Campbelltown, Australia; ^6^ Royal Prince Alfred Hospital, Camperdown, Australia, nsw.gov.au; ^7^ Department of Medicine and Therapeutics and Li Ka Shing Institute of Health Sciences, Faculty of Medicine, Prince of Wales Hospital, The Chinese University of Hong Kong, Sha Tin, Hong Kong, China, cuhk.edu.hk; ^8^ Shiv Nadar Institution of Eminence, Gautam Buddha Nagar, Uttar Pradesh, India; ^9^ Royal Brisbane and Women′s Hospital, Brisbane, Queensland, Australia

**Keywords:** biomarkers, diabetes, neutrophil elastase, OpenArray, qPCR

## Abstract

**Aims:**

The aim of the study is to integrate targeted transcriptomic analyses of previously identified biomarker proteins (proteomic findings) to better understand vascular complications (Cx) in Type 2 diabetes (T2D).

**Methods:**

Total RNA was extracted from baseline citrate plasma samples of 543 individuals with T2D from the Fenofibrate Intervention and Event Lowering in Diabetes (FIELD) trial. Among these, 224 participants had microvascular Cx, 142 had macrovascular Cx (51 had both types of Cx) and 228 had no Cx (control group). mRNA expression was quantified using the OpenArray platform and group differences were analysed using *ΔΔ*ct values. Neutrophil elastase (NE) protein levels were measured in baseline plasma by ELISA.

**Results:**

Eleven genes were retained for focused analysis. For microCx, the largest gene expression differences were observed for clusterin (~2‐fold upregulation) and integrin alpha‐IIb (~50% downregulation), compared with the control group. For macroCx, clusterin exhibited the strongest upregulation (~2.5‐fold), whereas apolipoprotein F showed the greatest downregulation (~20%). Comparative analysis of proteomic and transcriptomic data across study groups revealed that only 32% of gene expression differences were mirrored at the protein level. NE levels were highest in the microCx group and were significantly elevated versus control only in that group; NE levels also correlated inversely with circulating actin protein expression (*R* ^2^ = 0.92).

**Conclusion:**

Combining proteomic and targeted plasma transcriptomic data provides exploratory insights into biological processes associated with vascular complications in T2D and highlights NE as a candidate component of a proteolytic pathway for further investigation.

## 1. Introduction

Diabetes mellitus is a chronic metabolic disorder characterised by impaired glucose regulation resulting in persistent hyperglycemia [1]. The condition affects approximately 537 million people worldwide, with its prevalence steadily increasing and projected to reach 783 million by 2045 due to rising obesity rates and sedentary lifestyles [2]. Chronic diabetes can lead to macrovascular and microCx (including cardiovascular disease (CVD), stroke, myocardial infarction, amputation, retinopathy and nephropathy), significantly increasing morbidity [3] and mortality [4, 5] and presenting a high socioeconomic burden.

Advancing biomarker research is crucial for improving diabetes diagnosis, monitoring and treatment strategies. Traditional biomarkers, such as fasting plasma glucose, hemoglobin A1c, C‐peptide and HOMA‐scores are widely used to assess glycemic control and insulin sensitivity [6, 7]. Recently, advances in high‐throughput omics technologies have led to the discovery of novel biomarkers that can potentially improve diagnostic precision and predictive and prognostic capabilities over traditional biomarkers [8]. A recent study by Piarulli et al. that employed a targeted mass spectrometry approach identified 13 circulating proteins as potential biomarkers for progression of cardiovascular damage in individuals with T2D, demonstrating great classification results in terms of sensitivity and specificity [9]. Additionally, integrating multiomics data with machine learning algorithms has allowed the identification of robust signatures that may predict diabetic Cx, such as nephropathy [10] and retinopathy [11], with better accuracy than using a single biomarker.

In our previous proteomics study of Fenofibrate Intervention and Event Lowering in Diabetes (FIELD) trial participants [12, 13], we coupled large‐scale plasma proteomics with machine learning to identify 50 potential circulating protein biomarkers associated with T2D vascular Cx and to describe molecular pathways linked to these pathologies. In the present study, we used the cost‐effective, high‐throughput OpenArray platform to perform a targeted transcriptomic follow‐up of 48 of these candidates in FIELD participants′ baseline plasma, with the aim of assessing transcript detectability, comparing mRNA expression and protein levels directionality and exploring whether NE‐related signals warranted further investigation.

## 2. Research Design and Methods

### 2.1. Study Design

A detailed description of the FIELD study has been previously published [14]. In brief, the FIELD study was a double‐blinded, randomised, placebo‐controlled clinical trial designed to assess the impact of daily oral fenofibrate on cardiovascular health in individuals with T2D. The study enrolled 9795 participants in Australia, New Zealand and Finland. Ethics approval for the FIELD study and related substudies was obtained from the relevant Human Research Ethics Committees, and all participants provided written informed consent. The present biomarker analyses were conducted using stored baseline samples and associated deidentified clinical data in accordance with those approvals.

**Figure 1:**
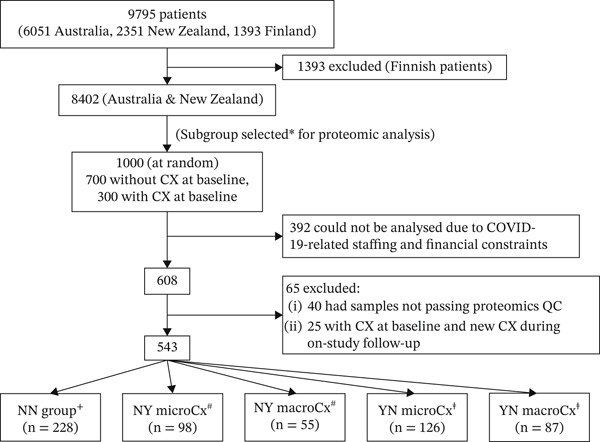
Derivation of the analytic sample for the present FIELD substudy. Microvascular and macrovascular analytic groups were complication‐specific rather than mutually exclusive; 51 participants had both complication types and could therefore contribute to both analytic arms.  ^∗^Matched for treatment allocation (fenofibrate vs. placebo) and key demographic and clinical characteristics including age, sex, ethnicity, diabetes duration, BMI, blood pressure, lipid profile, renal function, smoking status and glycaemic control; The symbols indicate: (+) no Cx at baseline—no Cx during on‐study follow‐up; (#) no Cx at baseline—Cx during on‐study follow‐up; (ǂ) Cx at baseline—No Cx during on‐study follow‐up.

In this targeted follow‐up substudy, we evaluated the mRNA expression of 48 of the 50 potential protein biomarkers previously identified as associated with T2D‐related vascular Cx [12, 13]. Current analysis was conducted within a previously defined, complication‐enriched sub‐study sample from the Australia/New Zealand FIELD cohort. Of the 1000 participants initially selected, 543 had available baseline citrate plasma and successfully completed biomarker analysis, after exclusion of participants with Cx present both at baseline and again during follow‐up (Figure 1). MicroCx were defined as history (self‐reported at baseline) or incidence (on‐study, validated) of having one or more of the following: peripheral neuropathy (abnormal monofilament test), nephropathy (urinary albumin‐to‐creatinine ratio ≥ 2.5 mg/mmol for men and ≥ 3.5 mg/mmol for women), retinopathy (on‐study laser treatment for diabetic retinopathy, including macular oedema) or amputation or minor (below ankle) amputation, without known peripheral vascular disease (PVD) in the ipsilateral limb.

Macrovascular complications (macroCx) were defined as history (self‐reported at baseline) or incidence (on‐study validated) of having one or more of the following: myocardial infarction, angina pectoralis, coronary artery bypass graft surgery, percutaneous transluminal coronary angioplasty, stroke, percutaneous revascularisation, intermittent claudication/PVD, amputation (with known PVD in the ipsilateral limb) or cardiovascular death.

Study participants were divided into groups according to the presence or absence of macroCx or microCx at two time‐points: baseline and during follow‐up, where “N” refers to absence of complication and “Y” refers to presence of complication at the respective timepoint:

1. No Cx at baseline—No Cx during on‐study follow‐up; reference group (NN) (*n* = 228).
2. MicroCx at baseline—No Cx during on‐study follow‐up; (YN microCx) (*n* = 126).
3. MacroCx at baseline—No Cx during on‐study follow‐up; (YN macroCx) (*n* = 87).
4. No Cx at baseline—MicroCx during on‐study follow‐up; (NY microCx) (*n* = 51).
5. No Cx at baseline—MacroCx during on‐study follow‐up; (NY macroCx) (*n* = 55).

These analytic groups were defined separately for microCx and macroCx analyses and were therefore not mutually exclusive. Participants who had at baseline or developed during on‐study follow up both complication types (*n* = 44) were included in both analytic arms. No formal matching strategy was used.

### 2.2. Proteomics

Sample preparation for proteomics analysis was performed as described earlier [15]. In brief, 1 *μ*L of sodium citrate plasma, collected at baseline, was added to 25 *μ*L of digestion buffer (100 mM Tris‐HCl, pH 8, 1% sodium deoxycholate, 10 mM tris(2‐carboxyethyl)‐phosphine, 40 mM chloroacetamide), heated at 95°C for 10 min, then diluted with 225 *μ*L of double‐distilled H_2_O containing 1 *μ*g of sequencing grade trypsin. Once digested, and peptides were recovered, all samples were run on an in‐house packed 150 *μ*m × 15 cm column, 1.9 *μ*m C18 LC column (ReproSil‐Pur C18‐AQ) over a 45 min gradient. Data independent acquisition (DIA) was conducted on a HF Orbitrap mass spectrometer using variable windows over the 300–1600 m/z range as described previously [15]. Spectra were analysed using Spectronaut Pulsar X (Biognosys) against our previously published spectral library [15], with precursor and protein Q‐value thresholds of 0.01. Peptides were quantified by a minimum of three transitions; protein quantification by the top *n* = 5 peptides. Exported matrices were then processed in Perseus V1.6.1.2 and log2 transformed. Not available (N/A) values were then imputed using values derived from the normal distribution reduced by three standard deviations.

### 2.3. RNA Extraction and Complementary DNA (cDNA) Synthesis

Total RNA was extracted from baseline plasma samples (200 *μ*L), using the Qiagen RNeasy 96 QIAcube HT Kit (QIAGEN, Hilden, Germany) according to the manufacturer′s protocol. Reverse transcription (RT) and cDNA synthesis were performed from 50 ng of RNA using the High‐Capacity cDNA RT Kit (Thermo Fisher Scientific, Waltham, Massachusetts). A synthetic spike‐in control of Arabidopsis thaliana miRNA (ath‐miR‐172a) was added to correct for any loss during the RNA isolation step. Ath‐miR‐172a RT‐PCR was performed using a miRNA‐specific RT enzyme and PCR primer‐probe mix (Thermo Fisher Scientific). cDNA was preamplified for 14 cycles with a custom OpenArray PreAmp Pool using a ViiA 7 system real‐time PCR (Thermo Fisher Scientific) before proceeding with qPCR using the OpenArray system.

### 2.4. OpenArray Technology

All material, accessories and reagents for OpenArray assay were sourced from Thermo Fisher Scientific and the procedure was carried out according to the manufacturer′s protocol. Because the current work was designed as a targeted follow‐up of prespecified proteomic candidates rather than an untargeted transcript discovery experiment, OpenArray was selected as a practical, high‐throughput platform for archived plasma samples. In brief, preamplified cDNA was diluted (1:20) and 2.5 *μ*L was loaded onto an OpenArray 384‐well sample plate containing 2.5 uL of TaqManOpenArray Real‐Time PCR Master Mix. The mixture was then transferred to a TaqMan OpenArray Real‐Time PCR Plate using the automated OpenArray AccuFill system. PCR plates were sealed and loaded into the QuantStudio 12 K Flex real‐time PCR instrument (qPCR, Thermo Fisher Scientific). A computer file containing PCR plate design, sample setup and thermo‐cycling conditions was uploaded prior to the qPCR run. For qPCR QC, we included a nontemplate control on every TaqMan OpenArray Real‐Time PCR plate and reviewed all OpenArray‐generated chip images to confirm between‐run consistency (dye signals, missed loading, etc.).

Selection of control genes for subsequent normalisation of the results was performed using an endogenous control plate (Thermo Fisher Scientific). A total of six genes were chosen for initial evaluation based on cycle threshold (ct) value and coefficient of variation. These controls included mitochondrially encoded ATP synthase 6 (MT‐ATP6), beta‐2‐microglobulin (B2M), peptidylprolyl isomerase A (PPIA), large ribosomal subunit protein uL10 (RPLP0), hydroxymethylbilane synthase (HMBS) and eukaryotic 18S rRNA (18S). MT‐ATP6 was selected empirically within this dataset as the best‐performing and most stable control in OpenArray (CV = 13.5*%*), with a detection rate ranging between 98% and 100% across the study groups (Table 1).

**Table 1:** Transcript detection rate of potential T2D biomarkers.

Gene	No. of ct values	Detection rate (%)	Min ct	Max ct
MT‐ATP6 ^∗^	538	99.47	13.25	29.94
18S rRNA ^∗^	515	94.84	10.62	30.33
ACTG1	491	90.42	21.71	33.60
ACTB	491	90.42	17.17	31.56
B2M ^∗^	478	88.03	18.35	30.87
RPLP0 ^∗^	437	80.48	23.10	31.16
PPIA ^∗^	418	76.98	23.79	30.61
PFN1	317	58.38	24.04	31.60
CLU	316	58.20	22.59	31.86
FERMT3	243	44.75	24.24	32.20
FLNA	231	42.54	24.55	33.04
GSN	172	31.68	24.78	34.37
ACTB1	147	27.07	24.76	32.58
TLN1	131	24.13	25.28	33.74
ITGA2B	128	23.57	23.45	31.88
APOF	127	23.39	26.44	32.02
CFP	64	11.79	26.83	34.54
CDH5	48	8.84	23.80	34.58
APOA2	47	8.66	27.69	31.84
FCN3	37	6.81	27.38	35.33
A2M	37	6.81	26.30	30.39
TPM2	34	6.26	29.99	32.90
LCAT	32	5.89	18.53	29.43
ARG1	25	4.60	28.46	31.73
APOC1	24	04.42	29.39	31.94
GPX3	22	4.05	28.33	32.00
C1QB	20	3.68	27.76	34.81
APOE	18	3.31	29.37	32.06
ITGA1	14	2.58	28.86	30.73
HMBS ^∗^	13	2.39	27.68	30.14
AGT	11	2.03	18.03	31.90
APOM	9	1.66	27.83	31.41
CFH	9	1.66	27.49	30.96
ITIH3	6	1.10	23.82	33.04
VWF	4	0.74	28.04	30.32
APOD	4	0.74	28.30	30.89
ECM1	3	0.55	30.94	33.17
CP	3	0.55	27.44	28.34
F5	3	0.55	18.37	30.54
SERPINA10	2	0.37	24.16	31.22
FGB	2	0.37	29.29	29.47
CPB2	2	0.37	28.71	30.31
TF	2	0.37	31.53	34.02
APOA1	2	0.37	28.84	29.74
AZGP1	2	0.37	29.92	31.05
F13B	1	0.18	30.96	30.96
FETUB	1	0.18	30.98	30.98
MBL2	0	0.00	—	—
PLG	0	0.00	—	—
KNG1	0	0.00	—	—
CPN1	0	0.00	—	—
MASP2	0	0.00	—	—
BCHE	0	0.00	—	—
LUM	0	0.00	—	—

*Note:* Genes marked with an asterisk ( ^∗^) indicate control genes.

### 2.5. NE Measurement

NE protein concentration was measured in baseline plasma samples using enzyme‐linked immunosorbent assay (ELISA) kits (Antibody and Immunoassay Services, University of Hong Kong, Hong Kong SAR) as described previously [16]. The intra‐ and inter‐assay coefficients of variation were < 8% and < 17%, respectively. All samples were analysed masked for subject identity, study treatment allocation and sample order.

### 2.6. Statistics and Pathway Analysis

qRT‐PCR data were analysed using the *ΔΔ*ct method to calculate relative gene expression fold changes [17]. Undetected ct values were replaced with a ct value of 40. An unpaired two‐tailed Student′s *t*‐test assuming unequal variances (Welch′s *t*‐test) was performed to calculate nominal statistical significance. Given the targeted exploratory nature of the transcriptomic analysis, *p* values were interpreted cautiously, and nonsignificant findings were considered as directional trends only.

Protein expression differences were determined by calculating fold changes as the ratio of mean label‐free quantification intensity in the complication groups versus the control group (YN vs. NN or NY vs. NN).

NE measurements were log10‐transformed for data normalisation. Statistical significance was assessed using Welch′s *t*‐test or one‐way ANOVA with significance defined as *p* < 0.05, and results are expressed as ng/mL with 95% CI.

Pathway analysis was conducted using the Database for Annotation, Visualisation and Integrated Discovery (DAVID; release v2024q2, 2024). Pathway findings were interpreted descriptively in the context of the previously published proteomic analysis.

## 3. Results

### 3.1. Transcriptomic Analysis

Out of 48 gene targets (representing our previously identified potential biomarkers), seven genes were below detection limit in all samples (i.e., mRNA not detectable in plasma), 13 genes had an average detection rate < 1% of samples, 18 genes had average detection rates between 1% and 19%, and 11 genes had average detection rates > 20% (Table 1). These 11 genes became the focus of our analysis. The detection rates for the 11 genes in each group are shown in Table 2.

**Table 2:** Transcript detection rate (%) of genes of interest (*n* = 11) per study group.

Gene	NN	YN macro	YN micro	NY macro	NY micro
ACTG1	89.5	95.4	90.5	89.1	89.8
ACTB	89.0	93.1	92.1	89.1	88.8
PFN1	56.1	64.4	56.3	60.0	55.1
CLU	51.8	60.9	61.9	63.6	62.2
FERMT3	43.4	46.0	47.6	52.7	39.8
FLNA	41.7	48.3	40.5	50.9	38.8
GSN	33.8	35.6	24.6	38.2	28.6
ITGA2B	23.7	24.1	23.0	32.7	17.3
TLN1	22.8	26.4	27.0	21.8	20.4
ACTN1	22.8	24.1	30.2	38.2	29.6
APOF	20.6	32.2	19.8	20.0	27.6

**Figure 2:**
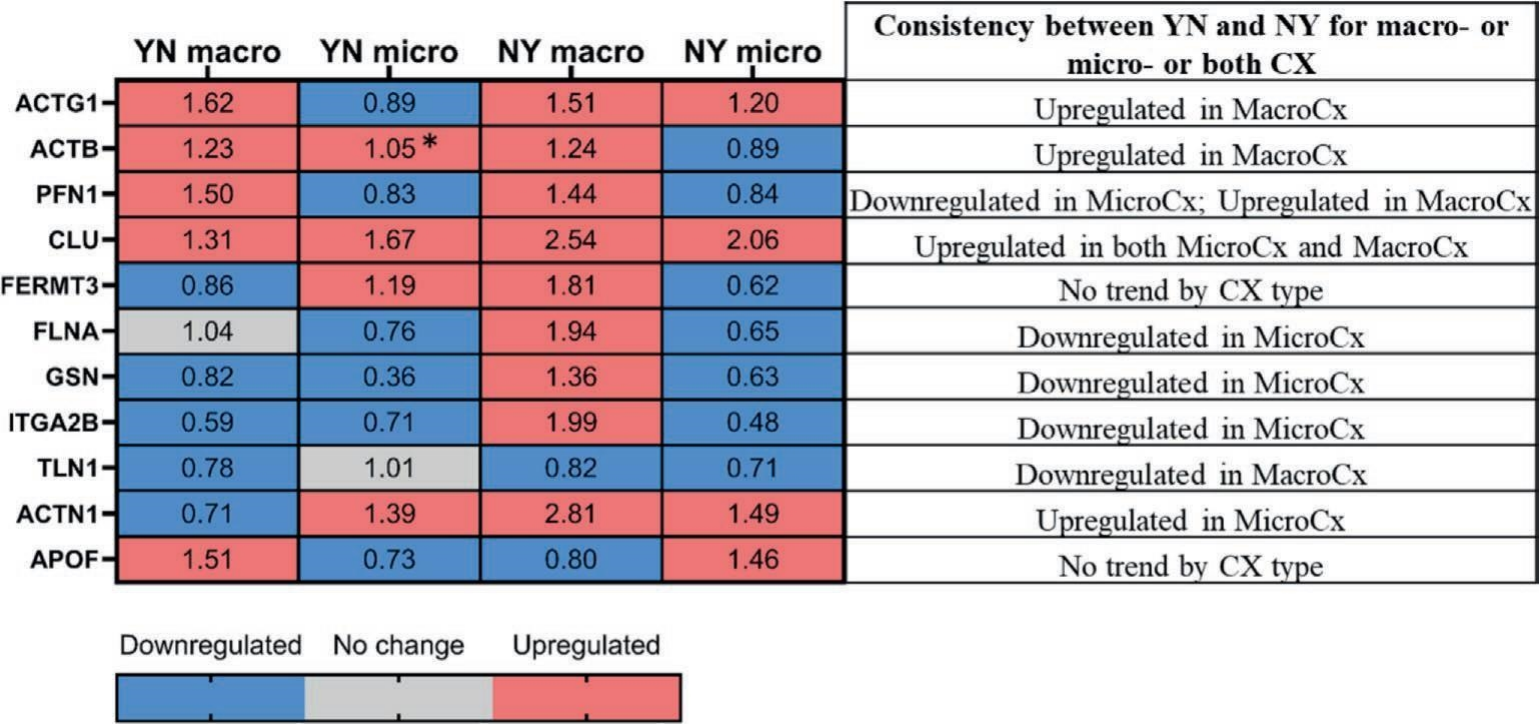
Gene expression fold changes (vs. NN group) and comments for genes of interest. Fold change > 1.05 implied upregulation; fold change < 0.95 implied downregulation; fold change that is within +/−5% range (0.95–1.05) was declared as no change. Colour coding was applied to facilitate visual interpretation of gene expression directionality across comparisons. Red shading indicates upregulated genes, blue shading indicates downregulated genes and grey shading indicates no substantial difference in expression  ^∗^Fold change for ACTB in YN micro was 1.052, hence why it is labelled as upregulated.

Gene expression, presented as fold change relative to the control group (NN), is summarised in Figure 2. The statistically significant transcript differences were; ITGA2B downregulation in NY microCx (~52% downregulation; *p* < 0.05) and GSN downregulation in YN microCx (~64% reduction; *p* < 0.01). Other differences, including CLU upregulation in NY microCx (~2‐fold; *p* = ns), ACTN1 upregulation in NY macroCx (~2.8‐fold; *p* = 0.054), CLU upregulation in YN microCx (~1.7‐fold; *p* = ns) and ACTG1 upregulation in YN macroCx (~1.6‐fold; *p* = 0.07), are reported only as directional trends and should be interpreted cautiously.

For descriptive purposes, a consistent gene expression pattern was defined as a gene exhibiting the same direction of fold change (i.e., either upregulation or downregulation) in both groups (NY and YN) of a complication type (macro‐ or microCx). The majority of genes (9 out of 11) demonstrated such consistency in at least one complication type. Notably, profilin 1 (PFN1) and CLU displayed concordant expression trends across both complication types. In contrast, fermitin family homolog 3 (FERMT3) and ApoF did not exhibit any consistent pattern (Figure 2).

### 3.2. Comparison of Transcriptomic and Proteomic Expression Data

**Figure 3:**
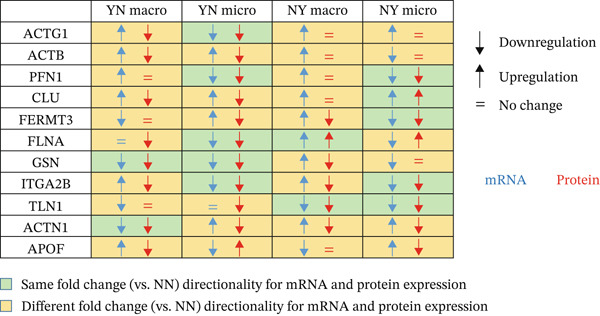
Comparison of mRNA and protein expression levels for protein candidates across study groups. Fold change values for mRNA (blue) versus the NN group and protein (red) versus the NN group are depicted as arrows indicating a difference in expression (above or below the 5% threshold) or an “=” symbol signifying to material difference versus NN group. Green cell shading indicates concordant fold‐change directionality between mRNA and protein expression, whereas yellow shading indicates discordant directionality.

To assess concordance between transcriptomic and proteomic data, we compared mRNA expression levels with corresponding protein abundance available from the published FIELD proteomics analysis [12, 13]. With 11 gene targets studied across four study groups (NN vs. NY microCx, YN microCx, NY macroCx, YN macroCx), there was a total of 44 instances where omics data were compared. In approximately 32% (14/44) of instances, mRNA expression exhibited the same directionality as protein expression (Figure 3). Most of these instances (12/14) belonged to the lower mRNA expression/lower protein expression category and were seen mainly in the microCx study groups; for example, downregulation of PFN1 mRNA expression in NY microCx coincided with downregulation of protein expression versus NN. A higher gene expression but equal or lower protein expression was observed in approximately 48% (21/44) of instances, consistent with posttranscriptional regulation, altered secretion or turnover, although these possibilities were not directly tested here. Only two targets, ITGA2B and PFN1, displayed consistent descriptive patterns across micro‐ and macroCx (NY and YN groups).

### 3.3. NE

To gain further insight into biological pathways associated with the 11 proteins retained for focused analysis, pathway enrichment analysis was performed using DAVID. Enriched pathways included focal adhesion (padj < 0.0001), regulation of the actin cytoskeleton (padj < 0.0001), platelet activation (padj < 0.0001) and neutrophil extracellular trap (NET) formation (padj < 0.05). Several proteins contributed to multiple enriched pathways, including ACTB and ITGA2B, which were represented across pathways related to cytoskeletal organisation, cell adhesion and platelet activation, indicating substantial functional overlap between the enriched biological processes. Proteins mapping to the NET formation pathway also overlapped with pathways involved in thrombo‐inflammatory signalling. Given the central role of NE in NET formation, circulating NE was subsequently assessed as a candidate component of a proteolytic pathway potentially relevant to vascular Cx in T2D.

**Figure 4:**
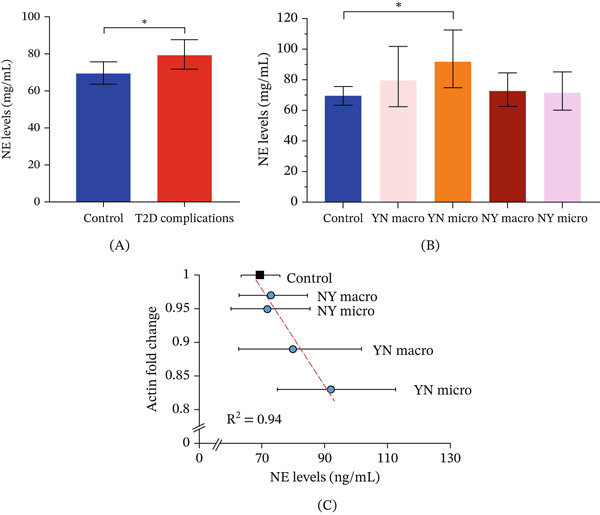
Neutrophil elastase (NE) levels across study groups and their correlation with ACTB expression. (A) Geometric mean (95% CI) of NE levels in NN (controls) and all Cx T2D groups (NY, YN, macroCx and microCx). For the purpose of statistical comparisons, NE values were log10‐transformed before performing an unpaired Student′s *t*‐test with Welch′s correction. (B) Geometric mean (95% CI) of NE levels in each group separately. NE levels, after log10 transformation, were compared across multiple groups using one‐way ANOVA. (C) Strong inverse correlation between NE and ACTB expression is observed (*R* ^2^ = 0.94). Geometric mean (95% CI) of NE levels is displayed for each group. The asterisk indicates statistical significance at the *p* < 0.05.

Elevated NE levels (vs. NN control group) were observed in the YN macroCx and YN microCx groups, whereas the NY macroCx (73 + 5.6/−5.2 ng/mL) and NY microCx (72 + 6.5/−6 ng/mL) groups had NE levels comparable to the NN control group (69 ± 3 ng/mL). The YN microCx group exhibited the highest NE levels (92 + 9.9/−8.9 ng/mL), followed by YN macroCx (80 + 10.2/−9.1 ng/mL) (Figure 4A,B). Only the comparison of YN microCx versus NN control was statistically significant (*p* < 0.05).

We then examined the relationship between NE levels and circulating actin (a recognised substrate of NE) protein levels. We observed a strong inverse correlation between NE concentration and circulating actin expression (*R* ^2^ = 0.94), which is consistent with, but does not prove, extracellular proteolytic activity in circulation (Figure 4C). The YN microCx group showed the lowest actin expression (−17%), followed by YN macroCx (−11%), NY microCx (−5%) and NY macroCx (−3%) versus the NN control group (Figure 4C).

## 4. Discussion

### 4.1. mRNA Detection in Plasma

We measured the mRNA expression of 48 proteins in FIELD trial participants′ plasma samples. These proteins were previously identified as associated with macrovascular and microvascular Cx of diabetes in our proteomics study [12, 13]. Only 11 genes had detectable transcripts in more than 20% of patients. Limited detection of mRNA in plasma is not unexpected, as circulating mRNA is generally present at very low concentrations under normal physiological conditions, unlike proteins, which are actively secreted or shed from cells [18]. The study of mRNA in circulation has been reported previously, but predominantly in cancer settings where cells actively release nucleic acids, including mRNA, into the bloodstream through mechanisms such as apoptosis, necrosis and active secretion via extracellular vesicles [19, 20]. In diabetes, to the best of our knowledge, there is only one report exploring mRNA presence in circulation, quantifying E2F transcription factor 1 levels in T2D [21]. The present work therefore provides an exploratory assessment of transcript detectability for previously identified proteomic candidates rather than comprehensive validation of plasma mRNA biomarkers. Low detectability may reflect low transcript abundance, instability of circulating RNA and the heterogeneous biological origin of plasma RNA. Alternative compartments, such as extracellular vesicle‐enriched fractions or cell‐associated RNA, may provide more comprehensive transcriptomic information in future studies. The current assay targeted RNA‐derived cDNA and was not designed to assess circulating DNA species.

### 4.2. Multiomics Expression Patterns

The comparison between transcriptomic and proteomic data revealed substantial variability in the concordance between directionality of mRNA expression and corresponding protein abundance across the Cx groups versus the NN group. Overall, mRNA and protein expression aligned in only 32% (14 out of 44) of instances, with the majority of concordant results displaying lower mRNA accompanied by reduced protein abundance, particularly within the microCx groups. This discordance does not invalidate the proteomic findings; rather, it underscores that plasma transcript abundance and plasma protein abundance are not expected to map one‐to‐one. One example is PFN1, which appears to be consistently repressed in the presence of microCx. Given that PFN1 plays a critical role in actin cytoskeletal remodelling [22], its downregulation in the presence of microCx may be relevant to endothelial dysfunction and vascular instability, both hallmark features of diabetes‐related microangiopathy. In contrast, nearly half of instances (21 out of 44) displayed increased mRNA expression without a corresponding increase in protein levels. One example is ITGA2B, which is primarily associated with platelet adhesion and thrombotic activity, and whose dysregulation may indicate altered responses in vascular remodelling or platelet function [23]. Such discordant patterns may reflect posttranscriptional regulation, reduced translation efficiency, altered secretion or increased protein turnover, although none of these mechanisms can be confirmed from the current data.

### 4.3. NE in Diabetes and Other Inflammatory Diseases

NE is a serine protease released during NET formation and is known to degrade proteins forming the extracellular matrix, tight junctions and cytoskeleton, including actin [24]. Although actin itself is not typically abundant in plasma under physiological conditions, reduced circulating actin levels may serve only as an indirect marker of extracellular NE‐related proteolysis. In our cohort, NE levels were highest in participants with established microvascular Cx, and the inverse relationship between NE concentrations and circulating ACTB protein levels is consistent with, but does not establish, extracellular NE activity in the circulation. This pattern was particularly pronounced in individuals with microvascular Cx, who exhibited the highest NE levels and the greatest reduction in ACTB expression. Higher NE levels in those with existing vascular Cx than in those who developed a complication during follow‐up may reflect a more chronic inflammatory state rather than incident risk per se [25, 26].

Similar observations have implicated NE in the pathogenesis of other chronic inflammatory diseases. For example, in conditions such as chronic obstructive pulmonary disease (COPD), cystic fibrosis and acute respiratory distress syndrome (ARDS), elevated extracellular NE has been linked to tissue injury and propagation of inflammation through degradation of substrate proteins in the extracellular matrix and cell junctions [27]. Moreover, NE′s extracellular activity has been the target of therapeutic intervention in some of these diseases, with inhibitors such as sivelestat and alvelestat showing the potential to reduce NE‐driven damage [28]. That precedents provide biological plausibility for further exploration of NE in diabetes Cx, although the chronic metabolic‐inflammatory milieu of T2D differs from these conditions and direct translation cannot be assumed.

### 4.4. Study Limitations

Although this exploratory study provides potential insights into the relationship between mRNA and protein expression in T2D‐related vascular Cx, several limitations should be considered. Detecting circulating mRNA in plasma presents technical and biological challenges, and the source of plasma mRNA is heterogeneous and not necessarily reflective of a single cell type, also reflecting varying levels of expression and clearance. mRNA may originate from immune cells, endothelial cells, platelets and even lysed red blood cells. The targeted candidate‐gene design and the low detection rate for many transcripts also limit the breadth of inference and statistical power. In addition, although our findings are consistent with a possible NE‐related pathway, we did not perform direct functional assays measuring NE enzymatic activity in plasma. The comparison structure in our study also limits causal inference and temporal interpretation, particularly because biomarker measurements were obtained at baseline and microvascular and macrovascular analytic groups were complication‐specific rather than mutually exclusive. Nevertheless, NE was reliably detectable in plasma, and the observed associations justify further study. Sequential measurements of NE and direct activity assays will be needed to determine whether elevated NE levels are causally related to T2D vascular Cx onset or progression.

## 5. Conclusion

This study is among the first to examine circulating plasma mRNA profiles in the context of T2D Cx and, to our knowledge, among the first to integrate these data with matched proteomic findings in a well‐characterised cohort with longitudinal clinical follow‐up. Our findings from this combined approach provide an exploratory view of biological processes associated with T2D‐related vascular Cx and generate new hypotheses regarding a potential role of NE in vascular injury in diabetes Cx.

## Author Contributions

Conceptualisation: H.F., A.S.J., A.A.H., A.J.J., M.P.M. and A.C.K. Methodology: H.F., A.S.M., M.L.H.H., F.K.E., A.A.H. and M.V.J. Formal analysis: H.F., A.S.J., A.S.M., M.E. and A.C.K. Investigation: H.F., A.S.M., F.K.E., A.A.H. and M.V.J. Data curation: H.F., A.S.J., A.S.M., M.E. and A.C.K. Visualisation: H.F., A.S.J., A.S.M. and M.E. Funding acquisition: A.A.H., R.C.W.M., S.G., V.G., M.E., A.J.J., M.P.M. and A.C.K. Project administration: A.S.J., A.J.J. and A.C.K. Supervision: A.S.J., A.J.J. and A.C.K. Writing—original draft: H.F., A.S.J., M.E., A.J.J., M.P.M. and A.C.K. Writing—review and editing: all authors. A.J.J., M.P.M. and A.C.K. have contributed to the work equally and should be regarded as co‐first authors.

## Funding

This study was supported by the National Health and Medical Research Council (1147897). Open access publishing facilitated by The University of Sydney, as part of the Wiley ‐ The University of Sydney agreement via the Council of Australasian University Librarians

## Disclosure

All authors have read and approved the final version of the manuscript. A.C.K. had full access to all of the data in this study and takes complete responsibility for the integrity of the data and the accuracy of the data analysis.

## Conflicts of Interest

The authors declare no conflicts of interest.

## Data Availability

Deidentified participant data may be made available upon reasonable request for academic use and within the limitations of the informed consent provided by the FIELD participants. Requests should be made to the corresponding author. Every request will be reviewed by representatives of the FIELD trial Steering Committee. Each researcher will need to sign a data access agreement with the National Health and Medical Research Council of Australia (NHMRC) Clinical Trials Centre of the University of Sydney after approval.
